# On Sulphuretted Hydrogen Gas as the Cause of Malarious Fevers

**Published:** 1843-09-16

**Authors:** 


### ON SULPHURETTED HYDROGEN GAS AS THE CAUSE OF MALARIOUS FEVERS.

*Extract from a letter of Dr. J. L. Day, Colonial Physician, dated Monrovia, July* 3, 1843, *to a distinguished Professor of Natural Philosophy, in the College of a neighbouring State.*

[Communicated by Professor DUNGLISON.]

"I cannot be too much obliged to you for the mark of your esteem in being at the pains to have copied for me the lengthy paper of Professor Daniell on the composition of the waters of this coast, &c. &c. He has scarcely left any room for one to make any reputation for himself on the track he has opened. He seems to have established conclusively in his own mind, that the insalubrity of this coast depends upon the excessive evolution of sulphuretted hydrogen, and adds: 'You have seen how constantly chlorine, [which he calls 'a certain remedy,'] destroys the gas.' 'Chlorine and sulphuretted hydrogen cannot exist together.' 'Plentiful fumigations of chlorine would therefore infallibly prevent,' &c. &c. And he alludes to the far-famed Niger expedition being fitted with means of constant chlorine fumigation;" and I may add, that when here we were shown this fumigating apparatus, by Captain Trotter, as a sort of life guard from the dreaded fever of this coast, and Professor Daniell's last words, after noticing this expedition, and the means of remedy adopted, are, 'We wait the result with anxiety.' And what has been the result? A most unexampled mortality, much greater than occurs in this settlement, where we are exposed on land to the miasm of the surrounding country, and where we have no fumigation of chlorine. Scarce men enough were left alive in the steamers to carry them home, and one of them was so completely disabled from sickness and death, when far up the river, that it would not have been enabled to get down but for the timely assistance of Captain Becroft, who had been once or twice up the river in a steamer of his own. I have not time to give you any more particulars ; but would ask, in conclusion, if Professor Daniell be now so sanguine that he has proposed 'a certain remedy.' "

